# Optimal control and cost-effective analysis of malaria/visceral leishmaniasis co-infection

**DOI:** 10.1371/journal.pone.0171102

**Published:** 2017-02-06

**Authors:** Folashade B. Agusto, Ibrahim M. ELmojtaba

**Affiliations:** 1 Department of Ecology and Evolutionary Biology, University of Kansas, Lawrence, 66045, Kansas, United States of America; 2 Department of Mathematics and Statistics, College of Sciences, Sultan Qaboos University, P.O.Box 36, Al Khodh, Oman; Academic Medical Centre, NETHERLANDS

## Abstract

In this paper, a deterministic model involving the transmission dynamics of malaria/visceral leishmaniasis co-infection is presented and studied. Optimal control theory is then applied to investigate the optimal strategies for curtailing the spread of the diseases using the use of personal protection, indoor residual spraying and culling of infected reservoirs as the system control variables. Various combination strategies were examined so as to investigate the impact of the controls on the spread of the disease. And we investigated the most cost-effective strategy of all the control strategies using three approaches, the infection averted ratio (IAR), the average cost-effectiveness ratio (ACER) and incremental cost-effectiveness ratio (ICER). Our results show that the implementation of the strategy combining all the time dependent control variables is the most cost-effective control strategy. This result is further emphasized by using the results obtained from the cost objective functional, the ACER, and the ICER.

## 1 Introduction

Malaria and visceral leishmaniasis (VL) are two major parasitic diseases with overlapping distributions which are both epidemiological and geographical in nature. This overlap may consequently lead to co-infection of the two parasites in the same patients [1]. Due to this co-infection, these parasites may partially share the same host tissues, with the ability to evade and subvert the host immune response; the clinical outcomes, however, depend largely on the immunological status of the host [1]. Furthermore, the success of the visceral *Leishmania donovani* complex obligate intracellular parasites in colonizing the macrophages and other reticulo-endothelial cells of the lymphoid system is due to their ability to alter the host’s parasite destruction signaling pathways and adaptive immunity engagement [2].

Visceral leishmaniasis patients who live in unstable seasonal malaria areas, such as eastern Sudan are exposed to the risk of co-infection [3]; however due to the variation in the geographical distribution of these co-infection cases, there might be some environmental and/or social factors associated with these risks of malaria-visceral leishmaniasis co-infections [3]. The prevalence of these co-infections in many VL’s endemic foci ranges from 31% in Sudan, 20% in Uganda and 1.2% in Bangladesh [3]. Concomitant malaria infections in unstable seasonal malaria areas are able to exacerbate VL symptoms in co-infected patients without affecting their prognosis if adequate and effective malaria treatment are provided; however, co-infected patients may experienced increase risks in mortality due to anti-malarial treatment failure to drugs such as chloroquine, sulfadoxine-pyrimethamine (SP) and quinine [3]. Hence, it is imperative for health officials in these VL foci with unstable malaria to ensure systematic malaria screening for all VL patients and artemisinin-based combination therapies (ACTs) treatment for patients with malaria [3].

Post-kala-azar dermal leishmaniasis (PKDL) occurs as a consequence of VL; it is caused by *leishmania donovani* in infected patients who have been cured of VL 6 months to 1 or more years prior to its appearance [4, 5]. It is common in VL endemic areas such as Sudan, Bangladesh, and India. PKDL may occur in endemic areas with *L. infantum* or *L. chagasi*, places such as the Mediterranean countries and Latin America [6]. *Leishmania donovani* in most cases is not a zoonotic parasite unlike *L. infantum*; however, there have been documentation of infected dogs in places with *L. donovani*. For instance Mo’awia *et al.* [7] showed that *phlebotomus orientalis* (VL main vector) in Sudan prefer dogs to other mammals like the Egyptian mongoose, common genet and Nile rat. Furthermore, domestic dogs might be the most important reservoir of *L. donovani* in eastern Africa [8, 9]. A study of VL risk factor in Ethiopia showed that dogs tested positive for VL antibodies [10]. Also, strains of *L. donovani* have been isolated from dogs in Kenya [11]. These studies iterates the possibilities of *L. donovani* being zoonotic with dogs as the reservoir, particularly in places like Ethiopia, Sudan and Kenya.

It is important to note that our study is on the model of malaria-visceral leishmaniasis co-infection, two infections that are endemic in Ethiopia, Sudan and Kenya. So without loss of generality we use this model to gain insight into understanding the dynamics of the co-infection. Thus, we have not incorporated any regional or parasite species specific features and parameters; these features will be incorporated as part of our future and further analysis. Thus, in this paper we propose an optimal control model for the dynamics of malaria-visceral leishmaniasis co-infection using the basic model of malaria-visceral leishmaniasis co-infection formulated in [12]. The aim of this work is to find the optimal and most cost-effective strategy to control both the mono-and co-infections in the community. This paper is organized as follows: in Section 2, we present the basic malaria-visceral leishmaniasis co-infection model and its main properties. In Section 3, we carry out a sensitivity analysis to identify the model’s parameters with the most impact on our response function. The optimal control problem is stated in Section 4 with some numerical simulation exploration carried out in Section 5. The cost-effectiveness analysis and discussions are given in Sections 6 and 7.

## 2 Malaria-visceral leishmaniasis co-infection model and its basic properties

In this study, we consider the model without control proposed and analyzed by Elmojtaba [12]. The model examined the dynamics of the malaria and visceral leishmaniasis co-infection in four populations; human host population *N*_*h*_(*t*), reservoir host population *N*_*r*_(*t*), mosquito population $N_{v_{m}}(t)$, and sandfly population $N_{v_{l}}(t)$. The human host population was divided into eight categories, individuals susceptible to both malaria and visceral leishmaniasis *S*_*h*_(*t*), those who are infected with malaria only $I_{h_{m}}(t)$, those who are infected with visceral leishmaniasis only $I_{h_{l}}(t)$, those who are infected with both malaria and visceral leishmaniasis $I_{h_{ml}}(t)$, The population also include those who have developed post kala-azar dermal leishmaniasis (PKDL) after the treatment of visceral leishmaniasis *P*_*h*_(*t*), those who have developed PKDL and have malaria $P_{h_{m}}(t)$, those who are recovered from leishmaniasis and have permanent immunity but susceptible to malaria *R*_*h*_(*t*) and those who are recovered from leishmaniasis and infected with malaria $R_{h_{m}}$. Hence, the total human population is given as

$$N_{h}\left(t\right)=S_{h}\left(t\right)+I_{h_{m}}\left(t\right)+I_{h_{l}}\left(t\right)+I_{h_{ml}}\left(t\right)+P_{h}\left(t\right)+P_{h_{m}}\left(t\right)+R_{h}\left(t\right)+R_{h_{m}}.\left(t\right)$$

The reservoir host population is divided into two categories, susceptible reservoir *S*_*r*_(*t*), and infected reservoir *I*_*r*_(*t*), such that the total population is

$$N_{r}\left(t\right)=S_{r}\left(t\right)+I_{r}\left(t\right).$$

The mosquito vector population is divided into two categories, susceptible mosquito vector $S_{v_{m}}(t)$, and malaria parasite infected mosquito vector $I_{v_{m}}(t)$, such that

$$N_{v_{m}}\left(t\right)=S_{v_{m}}\left(t\right)+I_{v_{m}}\left(t\right).$$

The sandfly population is similarly divided into two categories, susceptible sandflies $S_{v_{l}}(t)$, and VL parasite infected sandflies $I_{v_{l}}(t)$. Hence, the total population is

$$N_{v_{l}}\left(t\right)=S_{v_{l}}\left(t\right)+I_{v_{l}}\left(t\right).$$

It is assumed that susceptible humans are recruited into the population at a constant rate Γ. They acquire infection with malaria following contacts with infected mosquitoes at a per capita rate $a_{m}b_{m}\frac{I_{vm}}{N_{h}}$, where *a*_*m*_ is the per capita biting rate of mosquitoes on humans, and *b*_*m*_ is the transmission probability of malaria per bite per human. Furthermore, humans acquire infection with leishmaniasis following contacts with infected sandflies at a per capita rate $a_{l}b_{l}\frac{I_{vl}}{N_{h}}$, where *a*_*l*_ is the per capita biting rate of sandflies on humans (or reservoirs), and *b*_*l*_ is the visceral leishmaniasis transmission probability per bite per human. Humans infected with malaria acquire infection with leishmaniasis following contacts with infected sandflies at the same per capita rate as susceptible humans, die due to the disease at an average rate *δ*_1_ or recovered without immunity and became susceptible again at an average rate *γ*_1_.

Visceral leishmaniasis infected humans acquire infection with malaria following contacts with infected mosquitoes at the same per capita rate as susceptible humans, die due to leishmaniasis at an average rate *δ*_2_, or get treatment at an average rate *γ*_2_. A fraction *σ*_1_ of those who get treated recover and acquire permanent immunity, and the other fraction (1 − *σ*_1_) develop PKDL. Dually infected humans either recover from malaria and became VL only infected or get VL treatment and develop PKDL with malaria or recover from VL with malaria or die due to the co-infection at an average rate *δ*_3_, with the assumption that dual infection reduces both malaria recovery rate and VL treatment success.

Humans with PKDL only acquire infection with malaria following contacts with infected mosquitoes at the same per capita rate as susceptible humans, get treated at an average rate *γ*_3_, or recover naturally at an average rate *β* and acquire permanent immunity in both cases. Humans with PKDL and malaria get either PKDL treatment at an average rate *γ*_3_, or recover from PKDL naturally at an average rate *β* and acquire permanent immunity from VL in both cases, or recover from malaria and still suffer from PKDL. Humans who recovered from VL completely may acquire infection with malaria following contacts with infected mosquitoes at the same per capita rate as susceptible humans, and humans who recovered from VL completely and still suffer from malaria infection may recover from malaria infection at an average rate *γ*_1_, but they will not acquire any new VL infection. There is a per capita natural mortality rate *μ*_*h*_ in all human sub-population.

Susceptible reservoirs are recruited into the population at a constant rate Γ_*r*_, and acquire infection with leishmaniasis following contact with infected sandflies at a rate $a_{l}b_{l}\frac{I_{vl}}{N_{r}}$ where *a* and *b* as described above. We assume that the transmission probability per bite is the same for human and reservoir because sandflies do not distinguish between humans and reservoirs. It is also assumed that reservoirs disease induced death rate is negligible, but there is a per capita natural mortality rate *μ*_*r*_.

Susceptible mosquitoes are recruited at a constant rate $\Gamma_{v_{m}}$, and acquire malaria infection following contact with human infected with malaria, or humans dually infected, humans with PKDL and malaria or humans who recovered from all VL forms and infected with malaria with an average rate $a_{m}c_{m}\frac{\left(I_{h_{m}}+I_{h_{ml}}+P_{h_{m}}+R_{h_{m}}\right)}{N_{h}}$. Mosquitoes have a per capita natural mortality rate $\mu_{v_{m}}$ regardless of their infection status.

Susceptible sandflies are recruited at a constant rate $\Gamma_{v_{l}}$, and acquire leishmaniasis infection following contact with humans infected with leishmaniasis, humans dually infected, or human having PKDL (with or without malaria) or reservoir infected with leishmaniasis at an average rate of $a_{l}c_{l}[\frac{I_{h_{l}}+I_{h_{ml}}+P_{h}+P_{h_{m}}}{N_{h}}+\frac{I_{r}}{N_{r}}]$, it is also assumed that sandflies have a per capita natural mortality rate $\mu_{v_{l}}$ regardless of their infection status.

From the description above, we have the following system of differential equations representing the malaria-leishmaniasis co-infection:

$$S_{h}^{\prime }=\Lambda_{h}-a_{m}b_{m}I_{v_{m}}\frac{S_{h}}{N_{h}}-a_{l}b_{l}I_{v_{l}}\frac{S_{h}}{N_{h}}+\gamma_{1}I_{h_{m}}-\mu_{h}S_{h}$$ (1)

$$I_{h_{m}}^{\prime }=a_{m}b_{m}I_{v_{m}}\frac{S_{h}}{N_{h}}-a_{l}b_{l}I_{v_{l}}\frac{I_{h_{m}}}{N_{h}}-\left(\gamma_{1}+\delta_{1}+\mu_{h}\right)I_{h_{m}}$$

$$I_{h_{l}}^{\prime }=a_{l}b_{l}I_{v_{l}}\frac{S_{h}}{N_{h}}+\epsilon_{1}\gamma_{1}I_{h_{ml}}-a_{m}b_{m}I_{v_{m}}\frac{I_{h_{l}}}{N_{h}}-\left(\gamma_{2}+\delta_{2}+\mu_{h}\right)I_{h_{l}}$$

$$I_{h_{ml}}^{\prime }=a_{m}b_{m}I_{v_{m}}\frac{I_{h_{l}}}{N_{h}}+a_{l}b_{l}I_{v_{l}}\frac{I_{h_{m}}}{N_{h}}-\left(\delta_{3}+\epsilon_{1}\gamma_{1}+\epsilon_{2}\gamma_{2}+\mu_{h}\right)I_{h_{ml}}$$

$$P_{h}^{\prime }=\left(1-\sigma_{1}\right)\gamma_{2}I_{h_{l}}+\epsilon_{3}\gamma_{1}P_{h_{m}}-a_{m}b_{m}I_{v_{m}}\frac{P_{h}}{N_{h}}-\left(\gamma_{3}+\beta +\mu_{h}\right)P_{h}$$

$$P_{h_{m}}^{\prime }=a_{m}b_{m}I_{v_{m}}\frac{P_{h}}{N_{h}}+\left(1-\sigma_{2}\right)\epsilon_{2}\gamma_{2}I_{h_{ml}}-\left(\epsilon_{3}\gamma_{1}+\epsilon_{4}\gamma_{3}+\epsilon_{4}\beta +\mu_{h}\right)P_{h_{m}}$$

$$R_{h}^{\prime }=\sigma_{1}\gamma_{2}I_{h_{l}}+\left(\gamma_{3}+\beta \right)P_{h}+\gamma_{1}R_{h_{m}}-a_{m}b_{m}I_{v_{m}}\frac{R_{h}}{N_{h}}-\mu_{h}R_{h}$$

$$R_{h_{m}}^{\prime }=a_{m}b_{m}I_{v_{m}}\frac{R_{h}}{N_{h}}+\sigma_{2}\epsilon_{2}\gamma_{2}I_{h_{ml}}+\left(\epsilon_{4}\gamma_{3}+\epsilon_{4}\beta \right)P_{h_{m}}-\left(\gamma_{1}+\mu_{h}\right)R_{h_{m}}$$

$$S_{r}^{\prime }=\Lambda_{r}-a_{l}b_{l}I_{v_{l}}\frac{S_{r}}{N_{r}}+\mu_{r}S_{r}$$

$$I_{r}^{\prime }=a_{l}b_{l}I_{v_{l}}\frac{S_{r}}{N_{r}}-\mu_{r}I_{r}$$

$$S_{v_{m}}^{\prime }=\Lambda_{v_{m}}-a_{m}c_{m}S_{v_{m}}\frac{\left(I_{h_{m}}+I_{h_{ml}}+P_{h_{m}}+R_{h_{m}}\right)}{N_{h}}-\mu_{v_{m}}S_{v_{m}}$$

$$I_{v_{m}}^{\prime }=a_{m}c_{m}S_{v_{m}}\frac{\left(I_{h_{m}}+I_{h_{ml}}+P_{h_{m}}+R_{h_{m}}\right)}{N_{h}}-\mu_{v_{m}}I_{v_{m}}$$

$$S_{v_{l}}^{\prime }=\Lambda_{v_{l}}-a_{l}c_{l}S_{v_{l}}\left[\frac{I_{h_{l}}+I_{h_{ml}}+P_{h}+P_{h_{m}}}{N_{h}}+\frac{I_{r}}{N_{r}}\right]-\mu_{v_{l}}S_{v_{l}}$$

$$I_{v_{l}}^{\prime }=a_{l}c_{l}S_{v_{l}}\left[\frac{I_{h_{l}}+I_{h_{ml}}+P_{h}+P_{h_{m}}}{N_{h}}+\frac{I_{r}}{N_{r}}\right]-\mu_{v_{l}}I_{v_{l}}$$

with

$$N_{h}^{\prime }=\Lambda_{h}-\mu_{h}N_{h}-\left(\delta_{1}I_{h_{m}}+\delta_{2}I_{h_{l}}+\delta_{3}I_{h_{ml}}\right)$$

$$N_{r}^{\prime }=\Lambda_{r}-\mu_{r}N_{r}$$

$$N_{v_{m}}^{\prime }=\Lambda_{v_{m}}-\mu_{v_{m}}N_{v_{m}}$$

$$N_{v_{l}}^{\prime }=\Lambda_{v_{l}}-\mu_{v_{l}}N_{v_{l}}$$

The model variables and parameters are described in Tables 1 and 2.

**Table 1:** Description of the state variables of the co-infection model (1).

Variable	Description
*S*_*h*_(*t*)	Susceptible humans for both malaria and visceral leishmaniasis
$I_{h_{m}}(t)$	Malaria only infected humans
$I_{h_{l}}(t)$	Visceral leishmaniasis only infected humans
$I_{h_{ml}}(t)$	Co-infected humans
*P*_*h*_(*t*)	Humans who developed PKDL
$P_{h_{m}}(t)$	Humans who developed PKDL and infected with malaria
*R*_*h*_(*t*)	Humans who recovered from visceral leishmaniasis and susceptible to malaria
$R_{h_{m}}(t)$	Humans who recovered from visceral leishmaniasis and infected with malaria
*S*_*r*_(*t*)	Susceptible reservoirs
*I*_*r*_(*t*)	Infected reservoirs
$S_{v_{m}}(t)$	Susceptible mosquitoes
$I_{v_{m}}(t)$	Infected mosquitoes
$S_{v_{l}}(t)$	Susceptible sand flies
$I_{h_{l}}(t)$	infected sand flies

**Table 2:** Description of the parameters of the co-infection model (1).

Parameter	Description
Λ_*h*_	Humans recruitment rate
Λ_*r*_	Reservoirs recruitment rate
$\Lambda_{v_{m}}$	Mosquitoes recruitment rate
$\Lambda_{v_{l}}$	Sandflies recruitment rate
*μ*_*h*_	Natural mortality rate of humans
*μ*_*r*_	Natural mortality rate of reservoirs
$\mu_{v_{m}}$	Natural mortality rate of mosquitoes
$\mu_{v_{l}}$	Natural mortality rate of sandflies
*a*_*m*_	Biting rate of mosquitoes
*b*_*m*_	Progression rate of malaria in mosquito
*c*_*m*_	Progression rate of malaria in human
*a*_*l*_	Biting rate of sandflies
*b*_*l*_	Progression rate of VL in sandfly
*c*_*l*_	Progression rate of VL in human and reservoir
*u*_3_	Rate of recovery after treatment from malaria by humans
*u*_4_	Treatment rate of VL
*γ*_3_	PKDL recovery rate after treatment
1 − *σ*_1_	Developing PKDL rate after treatment
1 − *σ*_2_	Developing PKDL rate after treatment in co-infected
*δ*_1_	Malaria induced death rate
*δ*_2_	VL induced death rate
*δ*_3_	Co-infection induced death rate
*β*	PKDL recovery rate without treatment
*ϵ*_1_, *ϵ*_2_, *ϵ*_3_, *ϵ*_4_	Modification parameters

### Invariant region

All parameters of the model are assumed to be nonnegative, furthermore since model (1) monitors living populations, it is assumed that all the state variables are nonnegative at time *t* = 0. It is noted that in the absence of the diseases (*δ*_1_ = *δ*_2_ = *δ*_3_ = 0), the total human population size, *N*_*h*_ → Λ_*h*_/*μ*_*h*_ as *t* → ∞, also *N*_*r*_ → Λ_*r*_/*μ*_*r*_, $N_{v_{m}}\to \Lambda_{v_{m}}/\mu_{v_{m}}$ and $N_{v_{l}}\to \Lambda_{v_{l}}/\mu_{v_{l}}$ as as *t* → ∞. This shows that the biologically-feasible region:

$$\begin{array}{ccc} \Omega & = & \{\left(S_{h},I_{h_{m}},I_{h_{l}},I_{h_{ml}},P_{h},P_{h_{m}},R_{h},R_{h_{m}},S_{r},I_{r},S_{v_{m}},I_{v_{m}},S_{v_{l}},I_{v_{l}}\right)\in R_{+}^{14}:S_{h},I_{h_{m}},I_{h_{l}},I_{h_{ml}},\\ & & P_{h},P_{h_{m}},R_{h},R_{h_{m}},S_{r},I_{r},S_{v_{m}},I_{v_{m}},S_{v_{l}},I_{v_{l}}\geq 0,N_{h}\leq \frac{\Lambda_{h}}{\mu_{h}},N_{r}\leq \frac{\Lambda_{r}}{\mu_{r}},N_{v_{m}}\leq \frac{\Lambda_{v_{m}}}{\mu_{v_{m}}},N_{v_{l}}\leq \frac{\Lambda_{v_{l}}}{\mu_{v_{l}}}\} \end{array}$$

is positively-invariant domain, and thus, the model is epidemiologically and mathematically well posed, and it is sufficient to consider the dynamics of the flow generated by Eq (1) in this positively-invariant domain Ω.

The basic reproduction number is given by $R_{0}=\text{max}\{\sqrt{\frac{a_{m}^{2}b_{m}c_{m}\;m}{\mu_{v_{m}}\left(\gamma_{1}+\delta_{1}+\mu_{h}\right)}}\;,\;\sqrt{\frac{a_{l}c_{l}[\mu_{r}a_{l}b_{l}\;n\left(\gamma_{3}+\beta +\mu_{h}+\left(1-\sigma_{1}\right)\gamma_{2}\right)+a_{l}b_{l}\;k\left(\gamma_{2}+\delta_{2}+\mu_{h}\right)\left(\gamma_{3}+\beta +\mu_{h}\right)]}{\mu_{r}\mu_{v_{l}}\left(\gamma_{2}+\delta_{2}+\mu_{h}\right)\left(\gamma_{3}+\beta +\mu_{h}\right)}}\}$, and hence $R_{0}=\text{max}\{R_{m}\;,\;R_{l}\}$, where $R_{m},R_{l}$ are the reproduction numbers of malaria and leishmaniasis, respectively.

The following theorems summarize the important properties of the model (1), their proofs are given in [12].

**Theorem 2.1** *The* system (1) *has four equilibrium points:*

1. *The disease-free equilibrium, which is locally asymptotically stable if* $R_{0}$ *is less than unity, and globally stable if the conditions of either Lemma 3.2 or Lemma 3.3 of* [12] *satisfied*.
2. *The Visceral Leishmaniasis only endemic equilibrium, which is locally asymptotically stable if* $R_{l}$ *is greater than unity*.
3. *The Malaria only endemic equilibrium, which is locally asymptotically stable if* $R_{m}$ *is greater than unity*.
4. *The endemic equilibrium of coexistence, which exists if both* $R_{l}$ *and* $R_{m}$ *are greater than unity*.

**Theorem 2.2** *If the bifurcation quantity a is positive, then the* system (1) *undergoes a backward bifurcation which occurs at* $R_{0}=1$. *(i.e.* $R_{0}<1$ *is not sufficient for the eradication of the diseases.)*

## 3 Sensitivity analysis

Following [13, 14] we used the normalized forward sensitivity index also called elasticity, as it is the backbone of nearly all other sensitivity analysis techniques [15] and are computationally efficient [14]. The normalized forward sensitivity index of the quantity *Q* with respect to the parameter *θ* is given by:

$$S_{\theta }^{Q}=\frac{\partial Q}{\partial \theta }\times \frac{\theta }{Q}$$ (2)

Using the elasticity formula (2) and the parameter sets in Table 3, we now obtain numerical values for the elasticities. For each parameter *θ* we calculated the elasticity index of $R_{\prime }^{c}$ with respect to *θ*. Results are displayed in Table 4

**Table 3:** Parameters values of the co-infection model (1).

Parameter	Value	References
Λ_*h*_	0.0015875 × *N*_*h*_	[16]
Λ_*r*_	0.0073 × *N*_*r*_	Assumed
$\Lambda_{v_{m}}$	$0.071\times N_{v_{m}}$	[16]
$\Lambda_{v_{l}}$	$0.299\times N_{v_{l}}$	[17]
*μ*_*h*_	0.00004	[18]
*μ*_*r*_	0.000274	Assumed
$\mu_{v_{m}}$	0.05	[19]
$\mu_{v_{l}}$	0.189	[17]
*a*_*m*_	0.75	[20]
*b*_*m*_	Variable	Assumed
*c*_*m*_	0.8333	[21]
*a*_*l*_	0.2856	[22]
*b*_*l*_	Variable	Assumed
*c*_*l*_	0.0714	[23]
*u*_3_	Variable	Assumed
*u*_4_	Variable	Assumed
*γ*_3_	0.033	[24]
1 − *σ*_1_	0.36	[24]
1 − *σ*_2_	0.77	Assumed
*δ*_1_	0.0003454	[25]
*δ*_2_	0.011	[26]
*δ*_3_	0.06	Assumed
*β*	0.00556	[24]
*ϵ*_1_, *ϵ*_2_, *ϵ*_3_, *ϵ*_4_	0.07, 0.04, 0.07, 0.01	Assumed

**Table 4:** Sensitivity Indexes of the model’s parameters with respect to $R_{0}$.

Parameter	Sensitivity Index
*a*_*m*_	1
*b*_*m*_	0.5
*c*_*m*_	0.5
*μ*_*vm*_	−0.5
*γ*_1_	-0.49
*δ*_1_	−0.00087
*μ*_*h*_	0.000099
*a*_*l*_	1
*b*_*l*_	0.5
*c*_*l*_	0.5
*γ*_2_	−0.014
*γ*_3_	−0.0083
*β*	−0.0014
*σ*_1_	−0.017
*δ*_2_	−0.004
*μ*_*r*_	−0.471
*μ*_*vl*_	−0.5

It is very clear from Table 4, *a*_*m*_ and *a*_*l*_, the biting rate has the highest sensitivity index (s index = 1), which indicates that any increase (decrease) by *k*% in *a*_*m*_ or *a*_*l*_ will be followed by an immediate increase (decrease) by *k*% in $R_{\prime }$. The immediate conclusion is that at the disease-free equilibrium the most effective control strategy is the vector control.

The second highest sensitivity index (s index = 0.5) is associated with *b*_*m*_, *b*_*l*_, *c*_*m*_ and *c*_*l*_, the progression rate of the malaria and leishmaniasis in hosts and vectors, respectively. These parameters are out of control, therefore we can not use them as control parameters. Death rate of vectors has a sensitivity index of −0.5 which suggests that any increase by *k*% in *μ*_*vm*_ or *μ*_*vl*_ will be accompanied by a decrease of $\frac{k}{2}\%$ in $R_{0}$, and vice versa, which supports our claim that vector control is the most effective control strategy. The sensitivity index of the treatment rate of malaria is −0.49, which indicates that to reduce $R_{0}$ we need to increase the treatment rate. The death rate of the reservoir is also important in reducing $R_{0}$ because it has sensitivity index of −0.471. The sensitivity indexes for the other parameters are very small (−0.1–0.0001), which indicate that they have no effect on $R_{\prime }$. Therefore, in conclusion, the most effective control strategy is a strategy that involves vector control either by reducing their biting rate or increasing their death rate.

## 4 Optimal control problem

Following the conclusion obtained from the sensitivity analysis, we introduce into the malaria-visceral leishmaniasis model (1) four time-dependent controls *u*_1_(*t*), *u*_2_(*t*), *u*_3_(*t*) and *u*_4_(*t*). These time-dependent controls represent the use of personal protection measures (*u*_1_(*t*) and *u*_2_(*t*)) such as the use of insecticide-treated nets, application of repellents or insecticides to skin or to fabrics and impregnated animal collars (particularly dogs) [27] and the use of windows and door screens to prevent both mosquitoes and sandflies bites both during the day and at night. Furthermore, the time-dependent control *u*_3_(*t*) represents the culling of infected reservoir animals (like dogs) and the control *u*_4_(*t*) represents the use of insecticides such as DDT, pyrethroids and residual spraying of dwellings and animal shelters [27] to kill the mosquitoes and sandflies. Thus, the malaria-visceral leishmaniasis model (1) with time-dependent control is given as:

$$S_{h}^{\prime }=\Lambda_{h}-\frac{a_{m}b_{m}\left(1-u_{1}\right)I_{v_{m}}S_{h}}{N_{h}}-\frac{a_{l}b_{l}\left(1-u_{1}\right)I_{v_{l}}S_{h}}{N_{h}}+\gamma_{1}I_{h_{m}}-\mu_{h}S_{h}$$

$$I_{h_{m}}^{\prime }=\frac{a_{m}b_{m}\left(1-u_{1}\right)I_{v_{m}}S_{h}}{N_{h}}-\frac{a_{l}b_{l}\left(1-u_{1}\right)I_{v_{l}}I_{h_{m}}}{N_{h}}-\left(\gamma_{1}+\delta_{1}+\mu_{h}\right)I_{h_{m}}$$

$$I_{h_{l}}^{\prime }=\frac{a_{l}b_{l}\left(1-u_{1}\right)I_{v_{l}}S_{h}}{N_{h}}+\epsilon_{1}\gamma_{1}I_{h_{ml}}-\frac{a_{m}b_{m}\left(1-u_{1}\right)I_{v_{m}}I_{h_{l}}}{N_{h}}-\left(\gamma_{2}+\delta_{2}+\mu_{h}\right)I_{h_{l}}$$

$$I_{h_{ml}}^{\prime }=\frac{a_{m}b_{m}\left(1-u_{1}\right)I_{v_{m}}I_{h_{l}}}{N_{h}}+\frac{a_{l}b_{l}\left(1-u_{1}\right)I_{v_{l}}I_{h_{m}}}{N_{h}}-\left(\delta_{3}+\epsilon_{1}\gamma_{1}+\epsilon_{2}\gamma_{2}+\mu_{h}\right)I_{h_{ml}}$$

$$P_{h}^{\prime }=\left(1-\sigma_{1}\right)\gamma_{2}I_{h_{l}}+\epsilon_{3}\gamma_{1}P_{h_{m}}-\frac{a_{m}b_{m}\left(1-u_{1}\right)I_{v_{m}}P_{h}}{N_{h}}-\left(\gamma_{3}+\beta +\mu_{h}\right)P_{h}$$

$$P_{h_{m}}^{\prime }=\frac{a_{m}b_{m}\left(1-u_{1}\right)I_{v_{m}}P_{h}}{N_{h}}+\left(1-\sigma_{2}\right)\epsilon_{2}\gamma_{2}I_{h_{ml}}-\left(\epsilon_{3}\gamma_{1}+\epsilon_{4}\gamma_{3}+\epsilon_{4}\beta +\mu_{h}\right)P_{h_{m}}$$

$$R_{h}^{\prime }=\sigma_{1}\gamma_{2}I_{h_{l}}+\left(\gamma_{3}+\beta \right)P_{h}+\gamma_{1}R_{h_{m}}-\frac{a_{m}b_{m}\left(1-u_{1}\right)I_{v_{m}}R_{h}}{N_{h}}-\mu_{h}R_{h}$$ (3)

$$R_{h_{m}}^{\prime }=\frac{a_{m}b_{m}\left(1-u_{1}\right)I_{v_{m}}R_{h}}{N_{h}}+\sigma_{2}\epsilon_{2}\gamma_{2}I_{h_{ml}}+\left(\epsilon_{4}\gamma_{3}+\epsilon_{4}\beta \right)P_{h_{m}}-\left(\gamma_{1}+\mu_{h}\right)R_{h_{m}}$$

$$S_{r}^{\prime }=\Lambda_{r}-\frac{a_{l}b_{l}\left(1-u_{2}\right)I_{v_{l}}S_{r}}{N_{r}}-\mu_{r}S_{r}$$

$$I_{r}^{\prime }=\frac{a_{l}b_{l}\left(1-u_{2}\right)I_{v_{l}}S_{r}}{N_{r}}-\mu_{r}I_{r}-u_{3}I_{r}$$

$$S_{v_{m}}^{\prime }=\Lambda_{v_{m}}-\frac{a_{m}c_{m}\left(1-u_{1}\right)\left(I_{h_{m}}+I_{h_{ml}}+P_{h_{m}}+R_{h_{m}}\right)S_{v_{m}}}{N_{h}}-\mu_{v_{m}}S_{v_{m}}-u_{4}S_{v_{m}}$$

$$I_{v_{m}}^{\prime }=\frac{a_{m}c_{m}\left(1-u_{1}\right)\left(I_{h_{m}}+I_{h_{ml}}+P_{h_{m}}+R_{M}\right)S_{v_{m}}}{N_{h}}-\mu_{v_{m}}I_{v_{m}}-u_{4}I_{v_{m}}$$

$$S_{v_{l}}^{\prime }=\Lambda_{v_{l}}-a_{l}c_{l}S_{v_{l}}\left[\frac{\left(1-u_{1}\right)\left(I_{h_{l}}+I_{h_{ml}}+P_{h}+P_{h_{m}}\right)}{N_{h}}+\frac{\left(1-u_{2}\right)I_{r}}{N_{r}}\right]-\mu_{v_{l}}S_{v_{l}}-u_{4}S_{v_{l}}$$

$$I_{v_{l}}^{\prime }=a_{l}c_{l}S_{v_{l}}\left[\frac{\left(1-u_{1}\right)\left(I_{h_{l}}+I_{h_{ml}}+P_{h}+P_{h_{m}}\right)}{N_{h}}+\frac{\left(1-u_{2}\right)I_{r}}{N_{r}}\right]-\mu_{v_{l}}I_{v_{l}}-u_{4}I_{v_{l}}$$

Thus, we want to find the optimal values ($u_{1}^{*},\;u_{2}^{*},\;u_{3}^{*}$ and $u_{4}^{*}$) that minimizes the cost objective functional *J*(*u*_1_, *u*_2_, *u*_3_, *u*_4_) where

$$\begin{array}{ccc} J(u_{1},\;u_{2},\;u_{3},\;u_{4}) & = & \int_{0}^{t_{f}}\{A_{1}I_{h_{m}}+A_{2}I_{h_{l}}+A_{3}I_{h_{ml}}+A_{4}P_{h}+A_{5}P_{h_{m}}+A_{6}I_{r}+A_{7}I_{v_{m}}+A_{8}I_{v_{l}}\\ & & +C_{1}u_{1}^{2}+C_{2}u_{2}^{2}+C_{3}u_{3}^{2}+C_{4}u_{4}^{2}\}\;dt, \end{array}$$ (4)

This performance specification involves the numbers of infected humans, reservoirs, mosquitoes and sandflies, along with the cost of applying the controls (*u*_1_(*t*), *u*_2_(*t*), *u*_3_(*t*) and *u*_4_(*t*)). The coefficients, *A*_*i*_, *C*_*j*_, *i* = 1⋯8, *j* = 1⋯4, are balancing cost factors and *t*_*f*_ is the final time. The control quadruple (*u*_1_(*t*), *u*_2_(*t*), *u*_3_(*t*) and *u*_4_(*t*)) are bounded, Lebesgue integrable functions [28, 29]. The goal is to find the optimal control, $u_{1}^{*},\;u_{2}^{*},\;u_{3}^{*}$ and $u_{4}^{*}$, such that

$$J\left(u_{1}^{*},\;u_{2}^{*},\;u_{3}^{*},\;u_{4}^{*}\right)=\min_{U}\left\{J\left(u_{1},\;u_{2},\;u_{3},\;u_{4}\right)\right\}$$ (5)

where the control set,

$$\begin{array}{ccc} U & = & \{\left(u_{1}\left(t\right),\;u_{2}\left(t\right),\;u_{3}\left(t\right),\;u_{4}\left(t\right)\right),\;u_{i}:\left[0,t_{f}\right]\to \;\left[0,1\right],\;\;i=1,\cdots 4,\\ & & \;\text{is}\;\text{Lebesgue}\;\text{measurable}\}, \end{array}$$

### Characterization of optimal controls

The necessary conditions that an optimal control quadruple must satisfy come from the Pontryagin’s Maximum Principle [30]. This principle converts Eqs (3) and (4) into a problem of minimizing pointwise a Hamiltonian *H*, with respect to the controls (*u*_1_, *u*_2_, *u*_3_, *u*_4_). First we formulate the Hamiltonian from the cost functional Eq (4) and the governing dynamics Eq (3) to obtain the optimality conditions.

$$\begin{array}{ccc} H & = & A_{1}I_{h_{m}}+A_{2}I_{h_{l}}+A_{3}I_{h_{ml}}+A_{4}P_{h}+A_{5}P_{h_{m}}+A_{6}I_{r}+A_{7}I_{v_{m}}+A_{8}I_{v_{l}}\\ & & +C_{1}u_{1}^{2}+C_{2}u_{2}^{2}+C_{3}u_{3}^{2}+C_{4}u_{4}^{2}+\sum_{i}\lambda_{i}g_{i}, \end{array}$$ (6)

where *i* = *S*_*h*_, $I_{h_{m}}$, $I_{h_{l}}$, $I_{h_{ml}}$, *P*_*h*_, $P_{h_{m}}$, *R*_*h*_, $R_{h_{m}}$, *S*_*r*_, *I*_*r*_, $S_{v_{m}}$, $I_{v_{m}}$, $S_{v_{l}}$, $I_{v_{l}}$ and *g*_*i*_ are the right-hand sides of the system (3). Furthermore, $\lambda_{S_{h}}$, $\lambda_{I_{h_{m}}}$, $\lambda_{I_{h_{l}}}$, $\lambda_{I_{h_{ml}}}$, λ_*M*_, $\lambda_{P_{h}}$, $\lambda_{P_{h_{m}}}$, $\lambda_{R_{h}}$, $\lambda_{R_{h_{m}}}$, $\lambda_{S_{r}}$, $\lambda_{I_{r}}$, $\lambda_{S_{v_{m}}}$, $\lambda_{I_{v_{m}}}$, $\lambda_{S_{v_{l}}}$, $\lambda_{I_{v_{l}}}$ are the associated adjoints for the states *S*_*h*_, $I_{h_{m}}$, $I_{h_{l}}$, $I_{h_{ml}}$, *P*_*h*_, $P_{h_{m}}$, *R*_*h*_, $R_{h_{m}}$, *S*_*r*_, *I*_*r*_, $S_{v_{m}}$, $I_{v_{m}}$, $S_{v_{l}}$, $I_{v_{l}}$. The system of adjoint equations is found by taking the appropriate partial derivatives of the Hamiltonian Eq (6) with respect to the associated state and control variables.

**Theorem 1** *Given an optimal control quintuple (* $u_{1}^{*},\;u_{2}^{*},\;u_{3}^{*},\;u_{4}^{*}$ *) and solutions* $S_{h}^{*},I_{h_{m}}^{*},I_{h_{l}}^{*},I_{h_{ml}}^{*},P_{h}^{*},P_{h_{m}}^{*},R_{h}^{*},R_{h_{m}}^{*},S_{r}^{*},I_{r}^{*},S_{v_{m}}^{*},I_{v_{m}}^{*},S_{v_{l}}^{*},I_{v_{l}}^{*}$ *of the corresponding state* system (3) *that minimizes* $J(u_{R}^{*},\;u_{X}^{*},\;u_{V}^{*})$ *over* U. *Then there exists adjoint variables* $\lambda_{S_{h}}$, $\lambda_{I_{h_{m}}}$, $\lambda_{I_{h_{l}}}$, $\lambda_{I_{h_{ml}}}$, λ_*M*_, $\lambda_{P_{h}}$, $\lambda_{P_{h_{m}}}$, $\lambda_{R_{h}}$, $\lambda_{R_{h_{m}}}$, $\lambda_{S_{r}}$, $\lambda_{I_{r}}$, $\lambda_{S_{v_{m}}}$, $\lambda_{I_{v_{m}}}$, $\lambda_{S_{v_{l}}}$, $\lambda_{I_{v_{l}}}$ *satisfying*

$$\begin{array}{c} -\frac{d\lambda_{i}}{dt}\;=\;\frac{\partial H}{\partial i} \end{array}$$ (7)

*and with transversality conditions*

$$\begin{array}{ccc} \lambda_{i}\left(t_{f}\right) & = & 0,\;\;where\;\;i=S_{h},I_{h_{m}},I_{h_{l}},I_{h_{ml}},P_{h},P_{h_{m}},R_{h},R_{h_{m}},S_{r},I_{r},S_{v_{m}},I_{v_{m}},S_{v_{l}},I_{v_{l}}. \end{array}$$ (8)

*Furthermore, the control quadruple* $\left(u_{1}^{*},\;u_{2}^{*},\;u_{3}^{*},\;u_{4}^{*}\right)$ *are given as*

$$\begin{array}{ccc} u_{1}^{*} & = & \min\left\{1,max[0,\;\frac{1}{2C_{1}N_{h}}(a_{m}b_{m}I_{vm}S_{h}\left(\lambda_{I_{hm}}-\lambda_{S_{h}}\right)+a_{l}b_{l}I_{vl}S_{h}\left(\lambda_{I_{hl}}-\lambda_{S_{h}}\right)\right.\\ & & \left.+a_{l}b_{l}I_{vl}I_{hm}\left(\lambda_{I_{hml}}-\lambda_{I_{hm}}\right)+a_{m}b_{m}I_{vm}I_{hl}\left(\lambda_{I_{hml}}-\lambda_{I_{hl}}\right)+a_{m}b_{m}I_{vm}R_{h}\left(\lambda_{R_{hm}}-\lambda_{R_{h}}\right)\right.\\ & & \left.+a_{m}b_{m}I_{vm}P_{h}\left(\lambda_{P_{hm}}-\lambda_{P_{h}}\right)+a_{m}c_{m}\left(I_{hm}+I_{hml}+P_{hm}+R_{hm}\right)S_{vm}\left(\lambda_{I_{vm}}-\lambda_{S_{vm}}\right)\right.\\ & & \left.+a_{l}c_{l}S_{vl}\left(I_{hl}+I_{hml}+P_{h}+P_{hm}\right)\left(\lambda_{I_{vl}}-\lambda_{S_{vl}}\right))]\right\}, \end{array}$$ (9)

$$u_{2}^{*}=\;\min\left\{1,max\left[0,\;\;\frac{a_{l}}{2C_{2}}\left(\frac{c_{l}S_{vl}I_{r}\lambda_{I_{vl}}-b_{l}I_{vl}S_{r}\lambda_{S_{r}}+b_{l}I_{vl}S_{r}\lambda_{Ir}-c_{l}S_{vl}I_{r}\lambda_{S_{vl}}}{N_{r}}\right)\right]\right\},$$

$$u_{3}^{*}=\text{min}\left\{1,max[0,\frac{I_{r}\lambda_{I_{r}}}{2C_{3}}]\right\},$$

$$u_{4}^{*}=\min\left\{1,max\left[0,\;\;\frac{I_{vl}\lambda_{I_{vl}}+S_{vm}\lambda_{S_{vm}}+I_{vm}\lambda_{I_{vm}}+S_{vl}\lambda_{S_{vl}}}{2C_{4}}\right]\right\}.$$

*Proof*. The existence of an optimal control is guaranteed using the result by Fleming and Rishel [31]. Thus, the differential equations governing the adjoint variables are obtained by the differentiation of the Hamiltonian function, evaluated at the optimal controls. Thus, the adjoint system can be written as,

$$\begin{array}{c} \begin{array}{cc} -\frac{d\lambda_{S_{h}}}{dt} & =\;\frac{\partial H}{\partial S_{h}},\;\;\;\;\lambda_{S_{h}}\left(t_{f}\right)=0,\\ & \cdots \\ -\frac{d\lambda_{R_{h_{m}}}}{dt} & =\;\frac{\partial H}{\partial R_{h_{m}}},\;\;\;\;\lambda_{R_{h_{m}}}\left(t_{f}\right)=0,\\ & \cdots \\ -\frac{d\lambda_{S_{r}}}{dt} & =\;\frac{\partial H}{\partial S_{r}},\;\;\;\;\lambda_{S_{r}}\left(t_{f}\right)=0,\\ & \cdots \\ -\frac{d\lambda_{S_{v_{m}}}}{dt} & =\;\frac{\partial H}{\partial S_{v_{m}}},\;\;\;\;\lambda_{S_{v_{m}}}\left(t_{f}\right)=0,\\ & \cdots \\ -\frac{d\lambda_{S_{v_{l}}}}{dt} & =\;\frac{\partial H}{\partial S_{v_{l}}},\;\;\;\;\lambda_{S_{v_{l}}}\left(t_{f}\right)=0,\\ & \cdots \\ -\frac{d\lambda_{I_{v_{l}}}}{dt} & =\;\frac{\partial H}{\partial I_{v_{l}}},\;\;\;\;\lambda_{I_{v_{l}}}\left(t_{f}\right)=0, \end{array} \end{array}$$

evaluated at the optimal controls and corresponding state variables, results in the stated adjoint systems (7) and (8). Furthermore, differentiating the Hamiltonian function with respect to the control variables in the interior of the control set U, where 0 < *u*_*i*_ < 1, *i* = 1⋯4, we have

$$\begin{array}{ccc} \frac{\partial H}{\partial u_{i}} & = & 0 \end{array}$$ (10)

Then solving for controls $\left(u_{1}^{*},\;u_{2}^{*},\;u_{3}^{*},\;u_{4}^{*}\right)$ result in the optimality conditions given as

$$\begin{array}{ccc} u_{1}^{*} & = & \frac{1}{2C_{1}N_{h}}(a_{m}b_{m}I_{vm}S_{h}\left(\lambda_{I_{hm}}-\lambda_{S_{h}}\right)+a_{l}b_{l}I_{vl}S_{h}\left(\lambda_{I_{hl}}-\lambda_{S_{h}}\right)\\ & & +a_{l}b_{l}I_{vl}I_{hm}\left(\lambda_{I_{hml}}-\lambda_{I_{hm}}\right)+a_{m}b_{m}I_{vm}I_{hl}\left(\lambda_{I_{hml}}-\lambda_{I_{hl}}\right)+a_{m}b_{m}I_{vm}R_{h}\left(\lambda_{R_{hm}}-\lambda_{R_{h}}\right)\\ & & +a_{m}b_{m}I_{vm}P_{h}\left(\lambda_{P_{hm}}-\lambda_{P_{h}}\right)+a_{m}c_{m}\left(I_{hm}+I_{hml}+P_{hm}+R_{hm}\right)S_{vm}\left(\lambda_{I_{vm}}-\lambda_{S_{vm}}\right)\\ & & +a_{l}c_{l}S_{vl}\left(I_{hl}+I_{hml}+P_{h}+P_{hm}\right)\left(\lambda_{I_{vl}}-\lambda_{S_{vl}}\right)), \end{array}$$ (11)

$$u_{2}^{*}=\;\frac{a_{l}}{2C_{2}}\left(\frac{c_{l}S_{vl}I_{r}\lambda_{I_{vl}}-b_{l}I_{vl}S_{r}\lambda_{S_{r}}+b_{l}I_{vl}S_{r}\lambda_{Ir}-c_{l}S_{vl}I_{r}\lambda_{S_{vl}}}{N_{r}}\right)$$

$$u_{3}^{*}=\frac{I_{r}\lambda_{I_{r}}}{2C_{3}},$$

$$u_{4}^{*}=\frac{I_{vl}\lambda_{I_{vl}}+S_{vm}\lambda_{S_{vm}}+I_{vm}\lambda_{I_{vm}}+S_{vl}\lambda_{S_{vl}}}{2C_{4}}.$$ (12)

Using the bounds on the controls, the characterization Eq (9) can be derived.

**Remark 1** *Due to the a priori boundedness of the state and adjoint functions and the resulting Lipschitz structure of the ODE’s, the uniqueness of the optimal control for small time (t_f_) was obtained. The uniqueness of the optimal control quadruple follows from the uniqueness of the optimality system, which consists of* Eqs (3) and (7), Eq (8) *with characterization* Eq (9). *The restriction on the length of time interval is to guarantee the uniqueness of the optimality system, the smallness in the length of time is due to the opposite time orientations of* Eqs (3), (7) and (8); *the state problem has initial values and the adjoint problem has final values. This restriction is very common in control problems (see* [28, 32–37]*).*

Next we discuss the numerical solutions of the optimality system, the corresponding optimal control and the interpretations from various cases.

## 5 Numerical illustrations

Numerical solutions to the optimality system comprising of the state eq (3), adjoint eq (7), control characterizations Eq (8) and corresponding initial/final conditions are carried out using the forward-backward sweep method (implemented in MATLAB) and using parameters set in Table 3. The algorithm starts with an initial guess for the optimal controls and the state variables are then solved forward in time using Runge Kutta method of the fourth order. Then the state variables and initial control guess are used to solve the adjoint equations Eq (7) backward in time with given final condition (8), employing the backward fourth order Runge Kutta method. The controls *u*_1_(*t*), *u*_2_(*t*), *u*_3_(*t*), *u*_4_(*t*) are then updated and used to solve the state and then the adjoint system. This iterative process terminates when the current state, adjoint, and control values converge sufficiently [38].

In this section, we use numerical simulations to support the analytical results previously established, and to provide examples about the dynamics of both diseases. We use the following initial conditions *N*_*h*_ = 500,000, *N*_*r*_ = 10,000, $N_{v_{m}}=30,000$ and $N_{v_{l}}=50,000$. Most of the parameters used were found in the literature as seen in Table 3. However; some are assumed such as reservoirs recruitment rate and the natural mortality rate of reservoirs which is assumed because there are many potential reservoirs for leishmaniasis (see [39]). The rate at which humans develop PKDL after treatment in co-infection cases is assumed because we were unable to find any literature reference to it; therefore, we assume it is greater than the rate in VL only humans.

The disease induced death rate in co-infected humans is also assumed due to lack of literature information on it. We similarly assumed it is greater than VL induced death rate in humans who are infected with VL only. This assumption is made on the premise that there are increased risks of mortality in co-infected patients, possibly due to inappropriate anti-malarial treatment and treatment failure [1, 3]. Individuals in malaria endemic regions are known to self-medicate on anti-malarial drugs [40–44]. For instance, in Ethiopia, Deressa *et al.* [42] found that out of 616 households, 17.8% individuals self-mediate at home while 46.7% visit health services after self-medicating at home. These individuals use mainly chloroquine and sulfadoxine-pyrimethamine. Kimoloi *et al.* [43] found in a cross-sectional community based study in Kenya, that 74% out of the 338 participants self-medicate on antimalarial drugs such as sulphadoxine/sulphalene-pyrimethamine; majority (about 70.3%) self-medicated on Artemisinin-based combination therapies (ACT). Similarly in Sudan, Awad *et al.* [40] found 43.4% of the 600 study households had self-medicated on antimalarials such as chloroquine and sulfadoxine-pyrimethamine.

Thus, to illustrate the effect of different optimal control strategies on the spread of disease in a population, we will consider the following combination of time-dependent controls making up four control strategies A-D:

Strategy A: combination of *u*_1_(*t*), *u*_2_(*t*), *u*_3_(*t*) and *u*_4_(*t*)
Strategy B: combination of *u*_1_(*t*), *u*_2_(*t*) and while setting *u*_3_(*t*) = *u*_4_(*t*) = 0;
Strategy C: combination of *u*_3_(*t*) and *u*_4_(*t*) while setting *u*_1_(*t*) = *u*_2_(*t*) = 0 and
Strategy D: combination of *u*_1_(*t*), *u*_2_(*t*), *u*_3_(*t*) while setting *u*_4_(*t*) = 0.

### Strategy A: Using all the control variables

**Fig 1:**
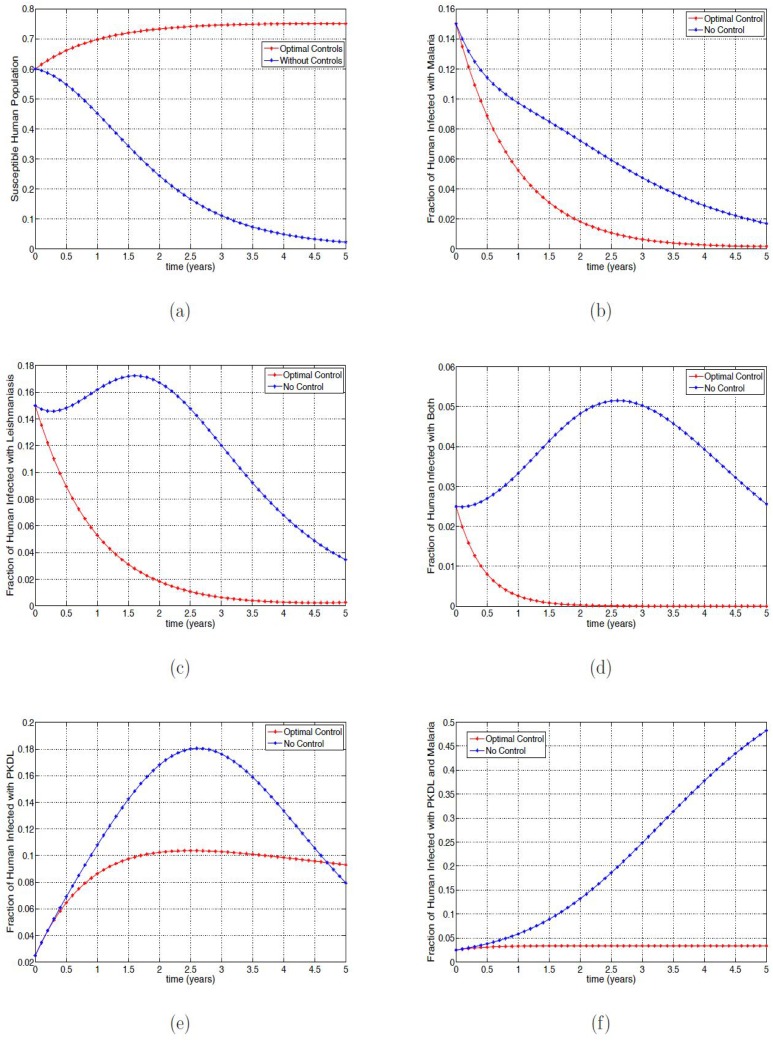
Simulation results of model 3 with and without controls. (a) Fraction of the susceptible humans; (b) Fraction of infected humans with VL; (c) Fraction of infected humans with malaria with and without controls; (d) Fraction of infected humans with malaria and VL. (e) Fraction of infected humans with PKDL; (f) Fraction of infected humans with PKDL and malaria.

Fig 1A shows the effect of applying all the optimal control (*u*_1_(*t*), *u*_2_(*t*), *u*_3_(*t*) and *u*_4_(*t*)) variables on the fraction of susceptible humans; without optimal controls over 70% of the population became infected within two years. However, when applying the controls only a small fraction of the population remain susceptible at the end of the simulation time. The optimal controls can be seen to result in a very small fraction of infected in the mono-infected classes (see Fig 1B and 1C as the controls act quickly from the onset of the application. Similar behavior is observed in Fig 1D, the co-infected class. The fraction of the co-infected individuals at the onset of the optimal controls quickly reduces, reaching zero co-infection in two years.

In Fig 1E, we observed that the fraction of infected humans with PKDL with and without optimal control were the same for about 3 months after which the fraction without control increased rapidly reaching the peak in about two and a half years compared to the fraction with optimal control which slightly rose and remained steady for four years seven months and then surpassing the fraction without during this time period. These infected individuals finally reach about 9.3%. In this same time period, the fraction without optimal control reduces to 7.9% compare those with control. This reduction is due to the fact that the fraction without control move quickly into the PKDL-Malaria co-infected class (see Fig 1F) after three and half years while those with control maintain a steady number with less than 5% co-infection.

**Fig 2:**
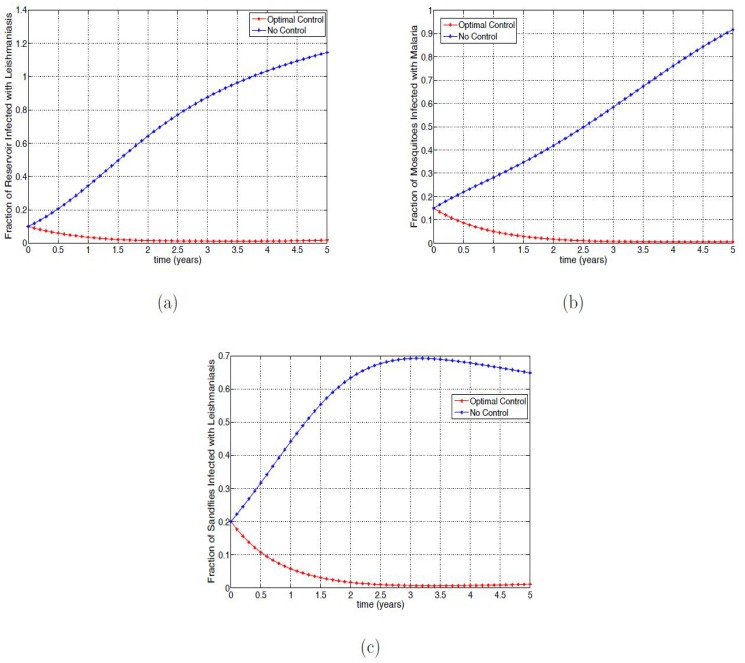
Simulation results of model 3 with and without controls. (a) Fraction of infected reservoir; (b) Fraction of infected mosquitoes with malaria; (c) Fraction of infected sandflies with VL.

Fig 2A, 2B and 2C shows the effect of the application of the optimal control on the fraction of the infected reservoir, mosquitoes, and sandflies; it is clear that applying the optimal controls reduce the fraction of these infected populations compared to the infected fraction without controls.

**Fig 3:**
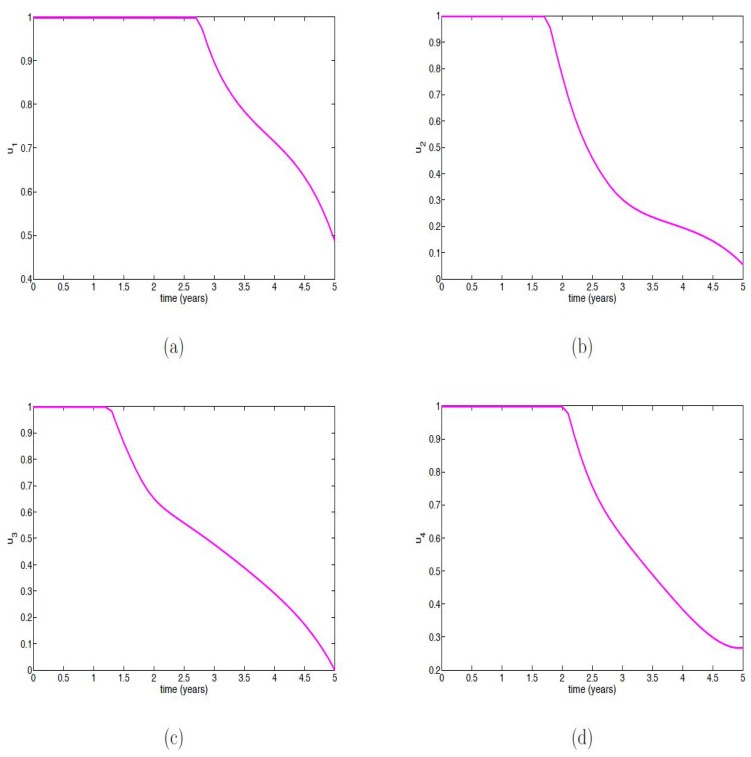
Simulation results of the control profile of model (3). (a) Control *u*_1_(*t*); (b) Control *u*_2_(*t*); (c) Control *u*_3_(*t*); (d) Control *u*_4_(*t*).

Fig 3 show the profiles of all the controls (*u*_1_, *u*_2_, *u*_3_ and *u*_4_) in which the optimal control is applied at the upper bound for about three, two, one and a half and two years, respectively; these are then reduced gradually until the end of the simulation period.

**Fig 4:**
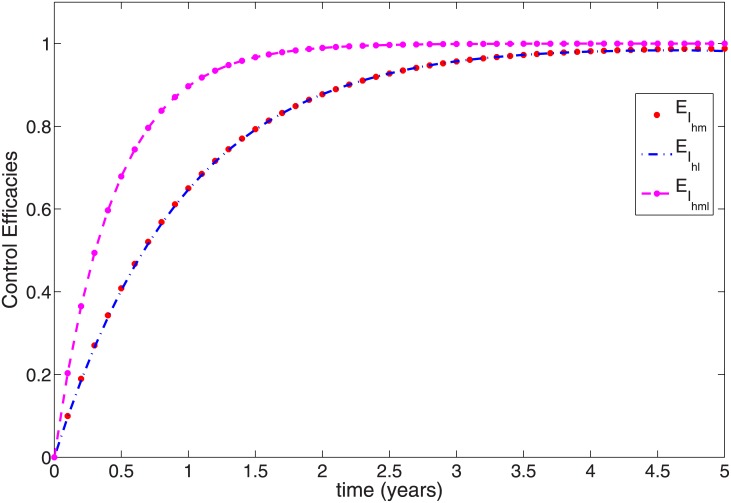
Efficacy function of the optimal control strategies of model (3).

To clearly show the efficacy of the control strategies in reducing the fraction of the infected with both mono-infection and co-infection, we follow the approach in [45] and define the efficacy function as

$$E_{I_{hm}}=\frac{I_{hm}\left(0\right)-I_{hm}^{*}\left(t\right)}{I_{hm}\left(0\right)},\;\;\;\;E_{I_{hl}}=\frac{I_{hl}\left(0\right)-I_{hl}^{*}\left(t\right)}{I_{hl}\left(0\right)},\;\;\;\;E_{I_{hml}}=\frac{I_{hml}\left(0\right)-I_{hml}^{*}\left(t\right)}{I_{hml}\left(0\right)},$$

where *I*_*hm*_(0), *I*_*hl*_(0), *I*_*hml*_(0) are the initial condition and $I_{hm}^{*}\left(t\right),\;I_{hl}^{*}\left(t\right),I_{hml}^{*}\left(t\right)$ are the fractions corresponding to the optimal state associated with the optimal controls $u_{1}^{*}\left(t\right),\;u_{2}^{*}\left(t\right),\;u_{3}^{*}\left(t\right)$ and $u_{4}^{*}\left(t\right)$. These functions measure the proportional decrease in the number of infected individuals caused by the intervention with optimal controls of strategies. The efficacy function depicted in Fig 4 indicates that adopting the optimal control strategies can reduce over 98% of infected individuals. The figure further shows that the control impact is quickest in the co-infected group with close to a 100% efficacy.

### Strategy B: Using personal protection measures for humans and the reservoirs

**Fig 5:**
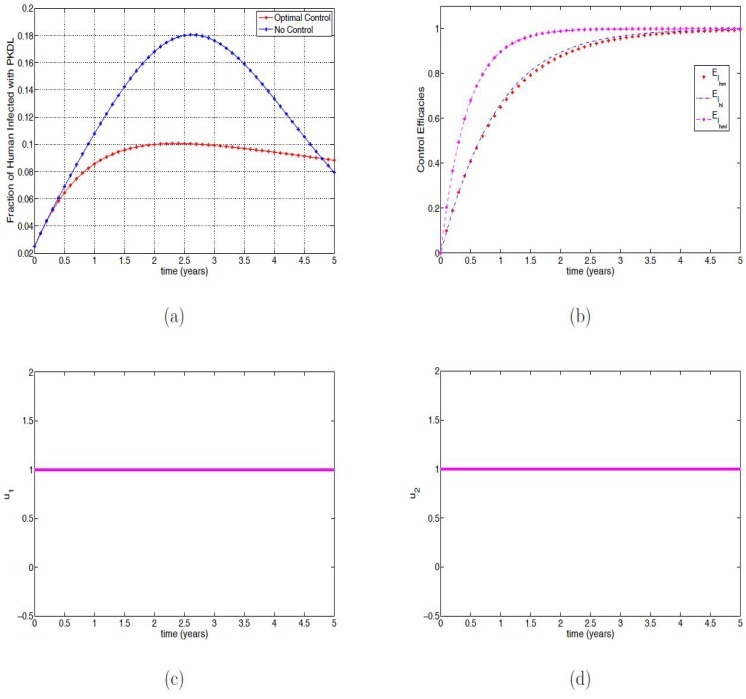
Simulation results of model (3) with and without controls. (a) Fraction of infected humans with PKDL; (b) Efficacy function of the optimal control strategies of model (3); (c) Control *u*_1_(*t*); (d) Control *u*_2_(*t*).

Strategy B, involving the use of personal protection measures for humans and the reservoirs (i.e. *u*_1_(*t*), *u*_2_(*t*) and while setting *u*_3_(*t*) = *u*_4_(*t*) = 0) has solution profiles that are similar to the profiles in Figs 1 and 2, except for Fig 1E and they are not shown here. In Fig 5A, we observed that the fraction of infected humans with PKDL with and without optimal control were the same for about 3.5 months after which the fraction without control increased rapidly reaching the peak in about two and a half years compared to the fraction with optimal control which also reaches the peak in two and a half years and reducing to about 9% infected individuals. Under this scenario, it takes about four years eight months for the trajectory of the with optimal control to surpass the trajectory of the without optimal control (compare Figs 1E and 5A). The efficacy function is depicted in Fig 5B and this indicates that adopting the optimal control strategies can reduce over 98% infected individuals with the control impact been quickest in the co-infected group with almost 100% efficacy. Fig 5C and 5D shows the optimal control profile which are at the upper bound for the two time-dependent controls (*u*_1_ and *u*_2_) employed.

### Strategy C: Using infected reservoir animal culling and indoor residual spraying

**Fig 6:**
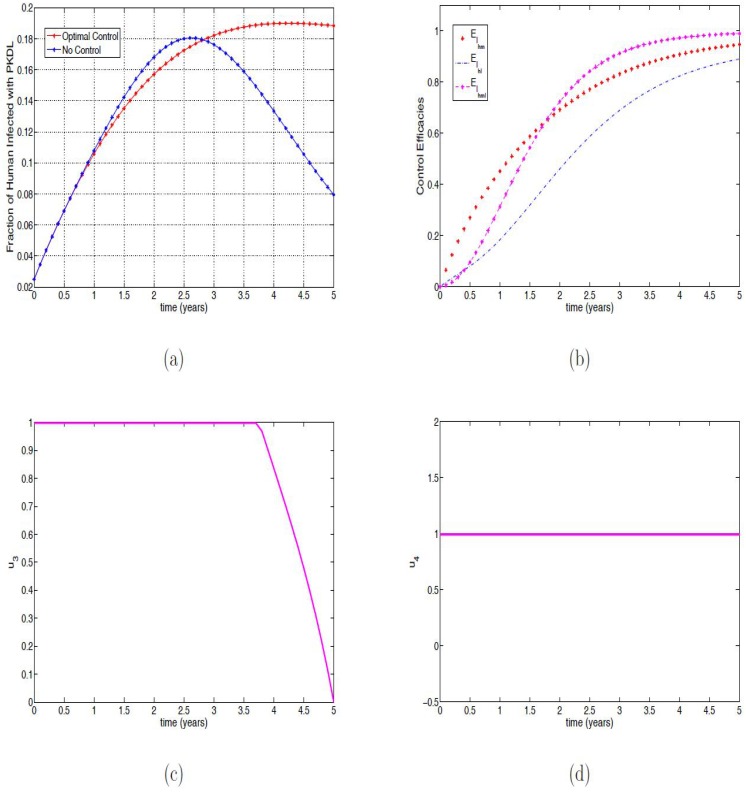
Simulation results of model (3) with and without controls. (a) Fraction of infected humans with PKDL; (b) Efficacy function of the optimal control strategies of model (3); (c) Control *u*_3_(*t*); (d) Control *u*_4_(*t*).

The solution profiles of Strategy C (i.e. *u*_3_(*t*) and *u*_4_(*t*) with *u*_1_(*t*) = *u*_2_(*t*) = 0) are also similar to the profiles in Figs 1 and 2, except for Fig 1E and are therefore not shown here as well. This control strategy involves the culling of infected reservoir animals and the use of insecticides such as DDT, pyrethroids, indoor residual spraying of human dwellings and animal shelters. In Fig 6A, we observed that the fraction of infected humans with PKDL with and without optimal control were the same for about six months after which the fraction without control slightly increased more than the fraction with optimal control, the infected fraction with control eventually surpassed the infected fraction without control after two years eight months. The PKDL infected fraction without control peaked at two years six months and then reduced to about 8% while the infected fraction with control remained at 18% (compare Figs 1E, 5A and 6A). This is due to the poor efficacy of the controls, the efficacy of the controls on malaria mono-infection is about 20% in the first five months while their impact on the leishmaniasis mono-infection and the co-infection is less than 10% in the same time period (see the efficacy function depicted in Fig 6B); although the control eventually had an efficacy of about 98% in the co-infection, about 94% in the malaria mono-infection and 89% in leishmaniasis mono-infection.

The efficacy of the control on malaria mono-infection and the co-infection reached 65% after a year eight months while leishmaniasis reached this performance level after about two years eight months (see Fig 6B). The low efficacy in the early period of the control implementation lead to this observed poor performance of this strategy even though the controls had to be maintained at very high levels, the control *u*_3_ was at the upper bound for about three years seven months before been gradually reduced, while the control *u*_4_ was kept at the upper bound throughout the simulation period. Fig 6C and 6D shows the optimal control profiles for these time-dependent control variables.

### Strategy D: Using personal protection measures and culling infected reservoirs

**Fig 7:**
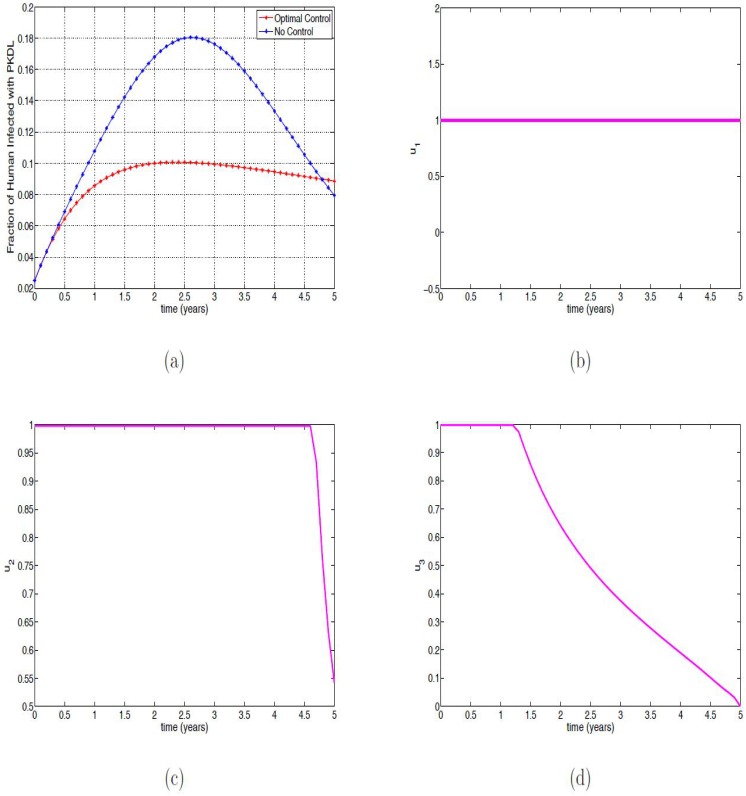
Simulation results of model (3) with and without controls. (a) Fraction of infected humans with PKDL; (b) Control *u*_1_(*t*); (c) Control *u*_2_(*t*); (d) Control *u*_3_(*t*).

**Fig 8:**
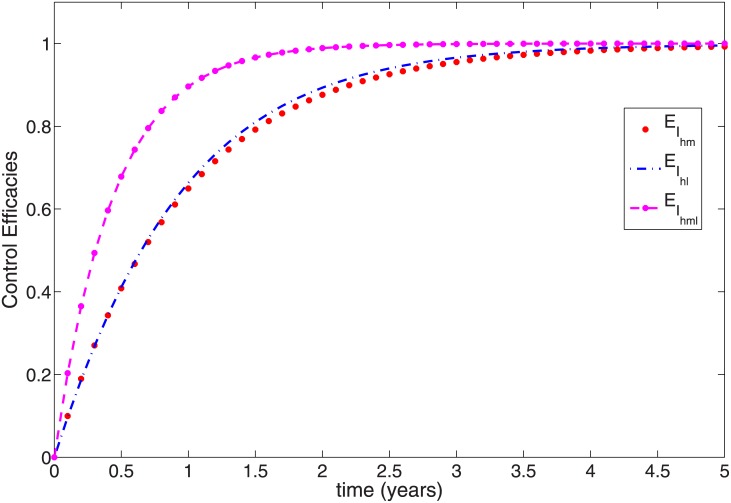
Efficacy function of the Optimal control strategies of model (3).

In utilizing this strategy (i.e., *u*_1_(*t*), *u*_2_(*t*), *u*_3_(*t*) while setting *u*_4_(*t*) = 0), we also observed similar profile as in Figs 1 and 2, except for Fig 1E and the rest are also not shown here. Thus, we observed in Fig 7A that it takes the PKDL fraction with optimal control four years eight months to surpass the fraction without control. After this time period, the PKDL fraction without optimal control reduced to about 8% while those with optimal control are at 8.8%. To maintain these efficacy, a lot of efforts is required of the three control variables (see Fig 7B–7D), the control *u*_1_ is expected to be at the upper bound throughout the simulation time period, while the control *u*_2_ is to be at the upper bound for four years six months and the control *u*_3_ is required to be at the upper bound for only a year and two months. This strategy has over 65% efficacy in the three controls in the less than a year of implementing the controls. Overall, it has over 99% efficacy in all the three controls (see Fig 8).

## 6 Cost-effectiveness analysis

Next, we performed a cost-effectiveness analysis. In order to justify the costs associated with health intervention(s) or strategy (strategies) such as treatment, screening, vaccination or educational intervention, the associated benefits are usually evaluated using cost-effectiveness analysis [32]. In this section we will consider three approaches, the infection averted ratio (IAR), the average cost-effectiveness ratio (ACER) and the incremental cost-effectiveness ratio (ICER).

### 6.1 Infection averted ratio

The infection averted ratio (IAR) is stated as:

$$\text{IAR}=\frac{\text{Number}\;\text{of}\;\text{infection}\;\text{averted}}{\text{Number}\;\text{of}\;\text{recovered}}.$$ (13)

**Fig 9:**
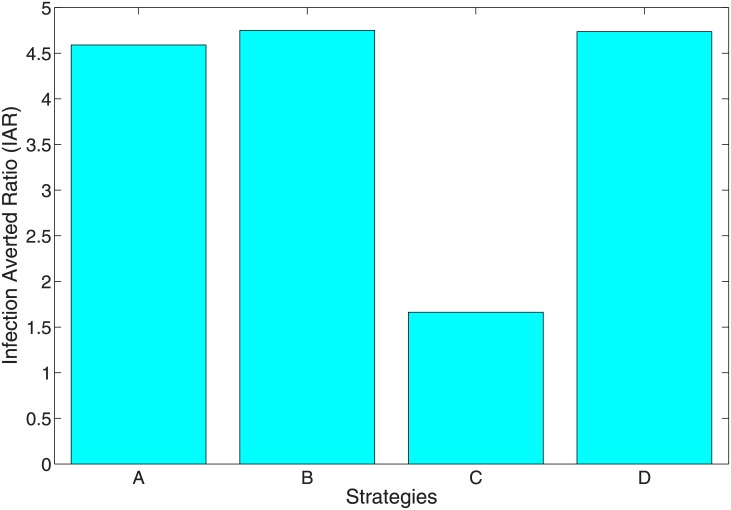
IAR plots indicating the effect of the control strategies A, B, C and D.

The number of infection averted above is given as the difference between the total infectious individuals without control and the total infectious individuals with control. The strategy with the highest ratio is the most effective. Using the parameter values in Table 3, the IAR for each intervention strategy was determined. Fig 9 shows the IAR for the four strategies implemented (see also Table 5). Strategy B involving the use of personal protection measures (*u*_1_(*t*) and *u*_2_(*t*) with *u*_3_(*t*) = *u*_4_(*t*) = 0) such as the use of insecticide-treated nets, application of repellents or insecticides to skin or to fabrics and impregnated animal collars produced the highest ratio and was therefore the most effective. This is followed by Strategy D involving the combination of personal protection measures (*u*_1_(*t*), *u*_2_(*t*)) and culling of infected reservoirs (*u*_3_(*t*)). The next effective strategy was Strategy A which combines all for control variables (*u*_1_(*t*), *u*_2_(*t*), *u*_3_(*t*) and *u*_4_(*t*)). Strategy C involving reservoir culling and insecticide use was the least effective, this in part was due to the low number of infection averted using this strategy (see Table 5).

**Table 5:** Total infection averted, total cost, IAR and ACER.

Strategies	Total infection averted	Total Cost	IAR	ACER
Strategy A	19.45382	823.0011	4.591037	42.30537
Strategy B	19.69121	1489.060	4.750906	75.62057
Strategy C	11.49082	1530.597	1.661732	133.2017
Strategy D	19.66483	1315.607	4.736452	66.90149

### 6.2 Average cost-effectiveness ratio (ACER)

Next, we considered the average cost-effectiveness ratio (ACER) which deals with a single intervention, evaluating it against the no intervention baseline option. ACER is calculated as

$$\text{ACER}=\frac{\text{Total}\;\text{cost}\;\text{produced}\;\text{by}\;\text{the}\;\text{intervention}}{\text{Total}\;\text{number}\;\text{of}\;\text{infection}\;\text{averted}}.$$ (14)

**Fig 10:**
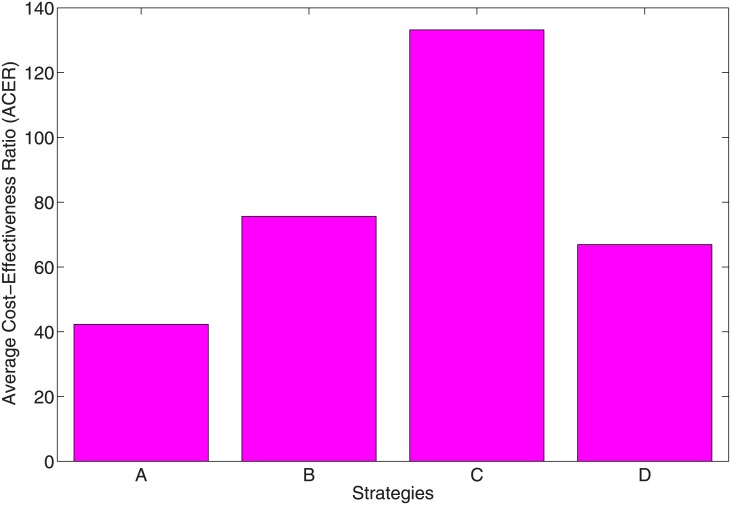
ACER plots indicating the effect of the control strategies A, B, C and D.

Fig 10 shows that the most cost-effective strategy is Strategy A, followed by Strategy D, then B. Strategy C is the least cost-effective (see also Table 5).

To further investigate the cost-effectiveness of the various control strategies, we evaluated the incremental cost-effectiveness ratio (ICER).

### 6.3 Incremental cost-effectiveness ratio

Disease control and eradication in a community can be both labor intensity and expensive. Thus, to determine the most cost-effective strategy to use, it is imperative to carry out a cost-effectiveness analysis. To achieve this, the differences between the various costs and health outcomes of implementing these different interventions are compared by calculating the incremental cost-effectiveness ratio (ICER). The ICER is the additional cost per additional health outcome and we assume that the costs of the various control interventions are directly proportional to the number of controls deployed. To compare competing intervention strategies (usually two or more) incrementally, one intervention is compared with the next-less-effective alternative [32]. Thus, the ICER is calculated as

$$\text{ICER}=\frac{\text{Difference}\;\text{in}\;\text{infection}\;\text{averted}\;\text{costs}\;\text{in}\;\text{strategies}\;i\;\text{and}\;j}{\text{Difference}\;\text{in}\;\text{total}\;\text{number}\;\text{of}\;\text{infection}\;\text{averted}\;\text{in}\;\text{strategies}\;i\;\text{and}\;j}.$$ (15)

The ICER numerator includes (where applicable) the differences in the costs of disease averted or cases prevented, the costs of intervention(s), and the costs of averting productivity losses among others. The ICER denominator on the other hand is the differences in health outcomes which may include the total number of infections averted or the number of susceptibility cases prevented.

To implement the ICER, we simulate the model using the various interventions strategies. Using these simulation results, we rank the control strategies in increasing order of effectiveness based on infection averted, we have that Strategy C averted the least number of infections, followed by Strategy A, Strategy D, and Strategy B which averted the most number of infections.

The ICER is computed as follows:

$$\text{ICER}(C)=\frac{1530.597}{11.4908}\;\;=\;\;133.2019$$

$$\text{ICER}(A)=\frac{823.0011-1530.597}{19.4538-11.4908}\;\;=\;\;-88.8605$$

$$\text{ICER}(D)=\frac{1315.607-823.0011}{19.6648-19.4538}\;\;=\;\;2334.6251$$

$$\text{ICER}(B)=\frac{1489.060-1315.607}{19.6912-19.6648}\;\;=\;\;6570.1894.$$

A look at Table 6, shows a cost saving of 6570.1894 for Strategy B over Strategy D, this is obtained by comparing ICER(D) and ICER(B). The lower ICER obtained for Strategy D is an indication that Strategy D strongly dominate Strategy B; this simply indicates that Strategy B is more costly to implement compare to Strategy D. Therefore, it is best to exclude Strategy B from the set of control strategies and alternative interventions to implement in order to preserve limited resources. Therefore, Strategy B is left out and Strategy D is further compared with Strategies A and C. Hence, we obtain the following numerical results in Table 7

**Table 6:** Incremental cost-effectiveness ratio in increasing order of total infection averted.

Strategies	Total infection averted	Total Cost	ICER
Strategy C	11.4908	1530.597	133.2019
Strategy A	19.4538	823.0011	−88.8605
Strategy D	19.6648	1315.607	334.6251
Strategy B	19.6912	1489.060	6570.1894

**Table 7:** Incremental cost-effectiveness ratio in increasing order of total infection averted.

Strategies	Total infection averted	Total Cost	ICER
Strategy C	11.4908	1530.597	133.2019
Strategy A	19.4538	823.0011	−88.8605
Strategy D	19.6648	1315.607	334.6251

Since ICER for strategies C and D are positive, their comparison shows a cost saving of 201.4181 for Strategy D over Strategy C. The lower ICER for Strategy C indicates that, Strategy C strongly dominate Strategy D. This implies that Strategy D will be more expensive to implement compare to Strategy C; thus, strategy D is excluded from further analysis. Hence, we obtain the following numerical computations given in Table 8 by excluding Strategy D and comparing the two remaining strategies, that is, Strategy A with C.

**Table 8:** Incremental cost-effectiveness ratio in increasing order of total infection averted.

Strategies	Total infection averted	Total Cost	ICER
Strategy C	11.4908	1530.597	133.2019
Strategy A	19.4538	823.0011	−88.8605

Table 8 shows a cost saving of 133.2019 for ICER(C) of Strategy C, over ICER(A) of Strategy A. Table 8 further indicate that Strategy A (from the negative ICER value) strongly dominate Strategy C. This simply implies that Strategy C is more costly and less effective compare to Strategy A. Thus, we exclude strategy C from further consideration.

Repeating the entire process, we can determine the next most cost-effective strategy. Thus, we found that Strategy D is the next cost-effective strategy after Strategy A, this is followed by Strategy B; Strategy C is the least cost-effective strategy and should be considered for implementation with a grain of salt.

**Fig 11:**
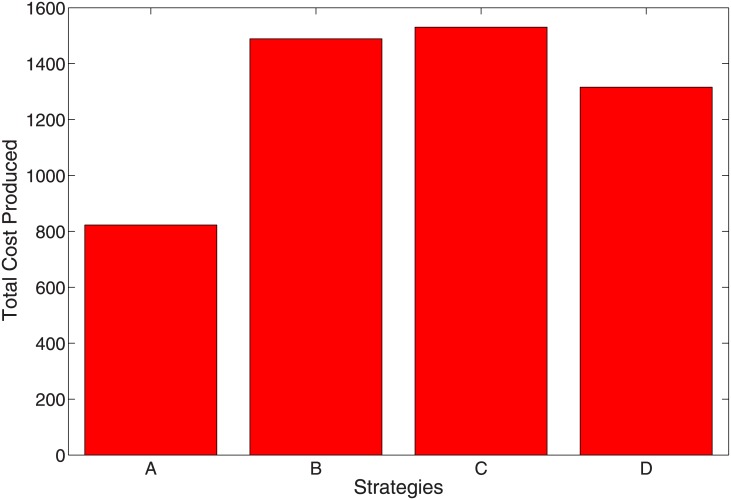
The objective functional indicating the effect of the control strategies A, B, C and D.

From this result, it is concluded that Strategy A (combination of all control variables *u*_1_, *u*_2_, *u*_3_ and *u*_4_) has the least ICER and therefore is more cost-effective than Strategy C. And is thus, the most cost-effective of all the strategies for control of both the mono infections and the co-infections. This result agrees with the results obtained in Fig 11 for the objective functional for the various control strategies.

## 7 Discussion and conclusion

In this paper, we applied optimal control theory to malaria/visceral leishmaniasis co-infection model developed in [12]. The analysis shows that the disease-free equilibrium of the model is locally asymptotically stable whenever the associated reproduction number ($R_{0}$), is less than unity and unstable otherwise. The model also exhibits backward bifurcation, a phenomenon where two stable equilibria coexist when the reproduction number is less than unity.

To identify the parameters with the strongest impact on the model outcome, in this case, the reproduction number, we used the normalized forward sensitivity index (elasticity). The results of the sensitivity analysis of the co-infection model show that the biting rates (*a*_*m*_ and *a*_*l*_) in both vectors have the highest sensitivity index. This is followed by the disease progression rates (*b*_*m*_, *b*_*l*_, *c*_*m*_ and *c*_*l*_) in hosts and vectors, respectively. The next strong impacting parameters are the death rate of vectors (*μ*_*vm*_ and *μ*_*vl*_). We also found that both the treatment rate and the death rate of the reservoirs have high negative sensitivity indexes, an indication that both parameters have a high impact in reducing $R_{0}$. However, our results show that control strategies that target vector and reduce contact with the vectors will be the most effective control strategy.

Thus, using these results from the sensitivity analysis, we introduced four time-dependent control variables into the model and investigated the associated benefits of different control strategies using cost-effectiveness analysis, so as to manage both the mono- and co-infections. This we did, using three approaches, the infection averted ratio (IAR), the average cost-effectiveness ratio (ACER) and the incremental cost-effectiveness ratio (ICER).

As expected, the control strategy utilizing all four control variables (Strategy A) is the most efficient strategy. This is not surprising, as this strategy involves the key parameters pertaining to vector reduction. Thus, this strategy reduces contact between humans and the two vectors (*via* the use of personal protection), reduces the two vector populations through the use of insecticide and reduces the infected reservoir population *via* culling. Other strategies (Strategies B and D) involving contact reduction *via* personal protection measures and reservoir culling are equally efficient, with efficacy as high as 98%. These high-efficiency levels in these strategies are due to an early on-start efficiency level which is as high as 65% within a year of implementing the controls. These early on-start efficiency level is lacking in Strategy C, which takes over a year and a half to reach the 65% efficacy level. This strategy eventually attains a high efficacy level (about 85%). This high efficacy level is due to the use of insecticide to reduce the vector populations which we know from our sensitivity analysis has a high negative impact on the reproduction number.

**Fig 12:**
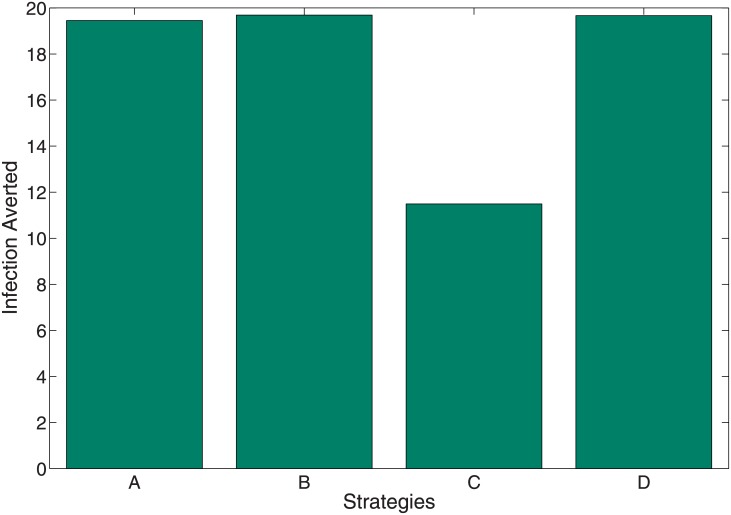
The objective functional indicating the effect of the control strategies A, B, C and D.

Following this results, it is therefore not surprising to see that Strategies A, B, and D averted the most number of infection, Strategy C performed the least (see Fig 12). This result linearly translates to the average cost, the ICER and objective functional and we can comfortably conclude, using the ICER result that Strategy A is the most cost-effective strategy to implement. This is followed by Strategy D, then B and Strategy C is the poorest and least effective strategy. It is the most costly if we are to follow the cost obtain from the average cost and objective functional (see Figs 10 and 11. Strategy C also averts the least number of infection.

In conclusion, malaria, and visceral leishmaniasis are two major parasitic diseases with tremendous negative consequences on the public health care system. In this paper, we have presented a deterministic model of a system of ordinary differential equations which couples the dynamics of malaria and visceral leishmaniasis co-infection. And we have studied using optimal control theory the use of personal protection, indoor residual straying and infected reservoir culling as effective control measures against the two co-infection epidemics. Therefore, the following results were observed from our analysis and numerical simulations:

1. The model has a DFE that is locally asymptotically stable if $R_{0}<1$;
2. The model also exhibit backward bifurcation, a phenomenon where two stable equilibrium coexist when the reproduction number is less than unity;
3. The application of time-dependent controls can reduce the total number of mono- and co-infected individuals in the population;
4. The most efficient and cost-effective control strategy is the strategy involving all the control variables (that is, Strategy A);
